# Sterility and Gene Expression in Hybrid Males of *Xenopus laevis* and *X. muelleri*

**DOI:** 10.1371/journal.pone.0000781

**Published:** 2007-08-22

**Authors:** John H. Malone, Thomas H. Chrzanowski, Pawel Michalak

**Affiliations:** Department of Biology, The University of Texas at Arlington, Arlington, Texas, United States of America; North Carolina State University, United States of America

## Abstract

**Background:**

Reproductive isolation is a defining characteristic of populations that represent unique biological species, yet we know very little about the gene expression basis for reproductive isolation. The advent of powerful molecular biology tools provides the ability to identify genes involved in reproductive isolation and focuses attention on the molecular mechanisms that separate biological species. Herein we quantify the sterility pattern of hybrid males in African Clawed Frogs (*Xenopus*) and apply microarray analysis of the expression pattern found in testes to identify genes that are misexpressed in hybrid males relative to their two parental species (*Xenopus laevis* and *X. muelleri*).

**Methodology/Principal Findings:**

Phenotypic characteristics of spermatogenesis in sterile male hybrids (*X. laevis* x *X. muelleri*) were examined using a novel sperm assay that allowed quantification of live, dead, and undifferentiated sperm cells, the number of motile vs. immotile sperm, and sperm morphology. Hybrids exhibited a dramatically lower abundance of mature sperm relative to the parental species. Hybrid spermatozoa were larger in size and accompanied by numerous undifferentiated sperm cells. Microarray analysis of gene expression in testes was combined with a correction for sequence divergence derived from genomic hybridizations to identify candidate genes involved in the sterility phenotype. Analysis of the transcriptome revealed a striking asymmetric pattern of misexpression. There were only about 140 genes misexpressed in hybrids compared to *X. laevis* but nearly 4,000 genes misexpressed in hybrids compared to *X. muelleri*.

**Conclusions/Significance:**

Our results provide an important correlation between phenotypic characteristics of sperm and gene expression in sterile hybrid males. The broad pattern of gene misexpression suggests intriguing mechanisms creating the dominance pattern of the *X. laevis* genome in hybrids. These findings significantly contribute to growing evidence for allelic dominance in hybrids and have implications for the mechanism of species differentiation at the transcriptome level.

## Introduction

Biological species remain cohesive by a lack of gene flow between interspecific populations and the mechanisms that maintain this pattern are manifest by various forms of reproductive isolation. Postzygotic reproductive isolation is characterized by dysfunctional phenotypes observed in F1 interspecific hybrids including but not limited to inviability and/or sterility resulting in decreased gene flow between species. Haldane (1922) [1] observed that the heterogametic sex (XY or ZW) typically suffers the most dysfunctional effects of hybridization. Haldane's rule has been shown to be broadly applicable across diverse groups of animals suggesting that understanding the basis for Haldane's rule provides a key towards understanding the mechanisms for how species become and/or remain reproductively isolated.

Much work, using mainly forward genetic approaches, has focused on the genetic basis of postzygotic reproductive isolation and Haldane's rule. These studies have provided support for both dominance effects and faster male evolution as the main mechanisms generating Haldane's rule; however only recently have studies focused on gene expression as related to sterile hybrids and this opens a new avenue towards understanding the proximate causes of reproductive isolation [2]–[12]. By starting at the level of phenotype, analyzing the transcriptome found in both species and compared to the dysfunctional hybrid, this reverse genetics approach provides the candidate loci that contribute to reproductive isolation, identifies targets that have evolved disparately between the two species, and allows a test of what evolutionary forces create gene misexpression in dysfunctional hybrids. Although correlational in nature, this approach advances our understanding of reproductive isolation by generating testable hypotheses for which future functional experiments can be designed.

Frogs of the genus *Xenopus* offer an exciting new system to explore the expression basis of reproductive isolation. *Xenopus* are characterized genomically by allopolyploidization and range from diploid (n = 20) to dodecaploid (n = 108) numbers of chromosomes [13]. Most species can be crossed to produce viable progeny and males from lab produced and wild caught hybrids are sterile whereas hybrid females are fertile [14]–[21]. Sex-reversal experiments and sex ratios from backcross progeny have established that a dominant allele in females of *X. laevis*, *X. muelleri*, and other species determines sex and therefore *Xenopus* have ZW sex determination [22]–[27]. Additionally, no morphologically distinct Z or W sex chromosomes have been identified in *Xenopus* and therefore *Xenopus* have homomorphic sex chromosomes [28], [29]. As *Xenopus* females are heterogametic and males are homogametic, yet in interspecies crosses the males are consistently sterile and females are fully or partially fertile, *Xenopus* provide an exception to Haldane's rule. Given the genetic peculiarities as well as the variety of genetic and genomic tools available for *Xenopus* as a model system, *Xenopus* offer an excellent system to address questions related to gene expression, reproductive isolation, and speciation.

We explored gene expression and reproductive isolation in hybrids of *Xenopus laevis* and *X. muelleri*, both tetraploid species (2n = 36) which hybridize in the wild [21]. To do so, we focused on the phenotypic characteristics of spermatogenesis in sterile male hybrids (*X. laevis* x *X. muelleri*). Affymetrix *Xenopus laevis* Genome Arrays were used to assay the transcriptome in testes combined with a correction for sequence divergence from genomic hybridizations that allowed discovery of the broad pattern of misexpression as well as the identification of candidate genes involved in the sterility phenotype. This approach importantly allows a correlation of the broad gene expression pattern to the phenotypic characteristics observed for sperm in sterile hybrids and identifies the loci misexpressed in hybrids relative to the two parental species.

## Results

### Sperm Abundance

One explanation for why male *Xenopus laevis* x *X. muelleri* are sterile could be due to phenotypic defects associated with the process of spermatogenesis. The production of amphibian sperm is a complex physiological process involving six key stages of differentiation that include primary spermatogonia, secondary spermatogonia, primary spermatocytes, secondary spermatocytes, and spermatids [30]. Additionally, it is widely accepted that the process of spermatogenesis is under hormonal control and that the injection of gonadotropins stimulates sperm production [31], [32]. If hybrid male *Xenopus* are sterile due to phenotypic defects associated with spermatogenesis, we predict that characteristics of sperm quality (i.e., abundance, motility, and morphology) should be different in hybrids compared to the parental species. We tested this hypothesis by injecting males with human chorionic gonadotropin hormone (hCG) to assay the effect of hormone induced stimulation on spermatogenesis in hybrids and the two parental species and then compared sperm characteristics of these injected males with uninjected sexually mature males.

Sperm abundance was quantified using a novel sperm assay that allowed detection of live, dead, and undifferentiated sperm cells. Testes were homogenized and then incubated with fluorescent dyes that intercalate with DNA of sperm cells based on whether the cell is live or dead. Counts of live, dead, and undifferentiated sperm cells were made using epifluorescence microscopy and we tested the null hypothesis that sperm abundance was the same in hybrids compared to the two parental species.

**Figure 1:**
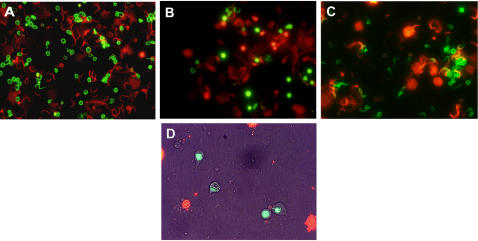
Visualization of live (green) and dead (red) sperm in sperm density assay using dual emission filter for SYBR14 and propidium iodide. *Xenopus laevis* (A); hybrid (B); *X. muelleri* (C); and brightfield combined with fluorescent image of undifferentiated cells of hybrids (D).

**Figure 2:**
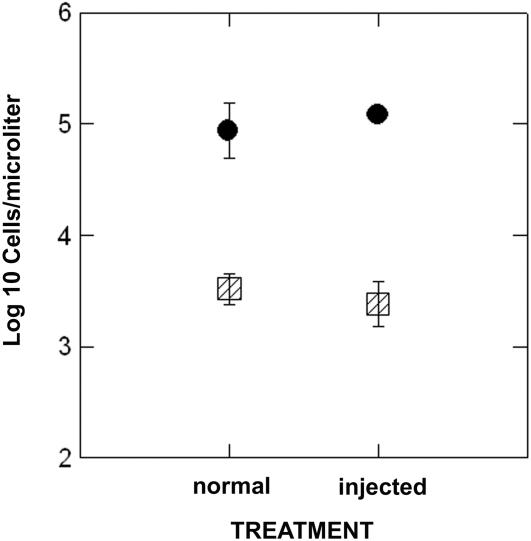
The number of total sperm cells in *Xenopus laevis* (black circles) and hybrids (striped squares) in uninjected sexually mature males and hCG injected sexually mature males. Error bars represent±1 standard error.

There was a dramatic difference in the abundance of sperm in *Xenopus laevis* compared to hybrids (Fig. 1a–c). About 40 times more sperm cells were found in *X. laevis* compared to hybrids (*F*_1,9_ = 135.4; *P* = 0.000) and there was no effect of the hCG treatment on the total number of sperm cells (*F*_1,9_ = 0.001; *P* = 0.978; Fig. 2). Only one *X. muelleri* was available for analysis and the abundance of sperm for this injected *X. muelleri* male (22,900 sperm/microliter) was comparable to the abundance of sperm for injected *X. laevis* (Mean sperm = 26,833 sperm/microliter) but not comparable to injected hybrids (Mean = 738 sperm/microliter).

**Figure 3:**
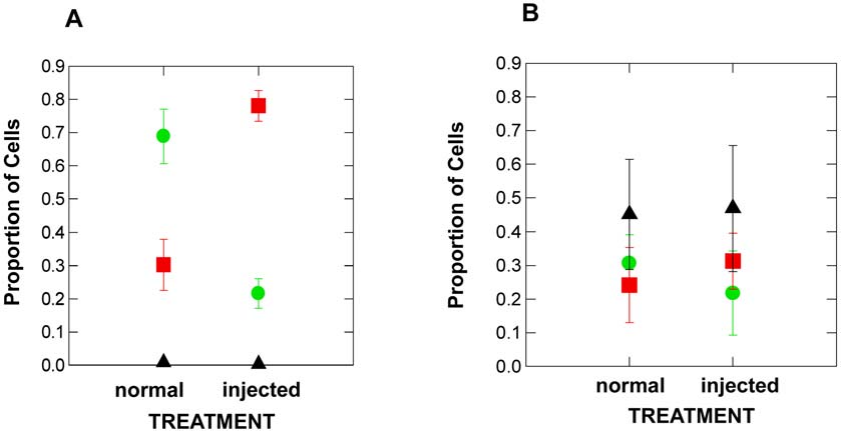
Proportion of live (green circles), dead (red squares), and undifferentiated (black triangles) sperm cells in *Xenopus laevis* (A) and hybrids (B) in normal/uninjected compared to hCG injected males. Error bars represent±1 standard error.

Proportions of live and dead sperm cells revealed an effect of hCG treatment on *Xenopus laevis* only. The proportion of live sperm cells was about 40% greater in normal *X. laevis* compared to hybrids (*F*_1,8_ = 7.13; *P* = 0.028) and there was an affect of hCG treatment on the proportion of live sperm cells (*F*_1,8_ = 15.75; *P* = 0.004). This hCG treatment effect can be explained mainly by the *X. laevis* specific response to hCG treatment (Taxa x Treatment interaction *F*_1,8_ = 6.31; *P* = 0.036) as the proportion of live cells decreased by about 50% (because the proportion of dead cells increased from 30% to 50% from the hCG treatment) in *X. laevis* but did not change in hybrids (Fig. 3). The proportion of live and dead cells for *X. muelleri* (Live = 0.52; Dead = 0.48) was higher compared to injected *X. laevis* (Mean proportion Live *X. laevis = *0.22; Mean proportion Dead *X. laevis = *0.78) but was closer to the mean proportion of live and dead cells for normal *X. laevis* (Mean proportion Live = 0.69; Mean proportion Dead = 0.30)

There was a striking difference in the proportion of undifferentiated sperm cells in *Xenopus laevis* compared to hybrids (Fig. 1b, d). In *X. laevis* there were practically no undifferentiated sperm cells but in hybrids the majority (about 50%) of the sperm suspension was comprised of undifferentiated sperm (*F*_1,8_ = 46.073; *P* = 0.000). The hCG treatment had no effect on the number of undifferentiated sperm cells (*F*_1,8_ = 0.013; *P* = 0.911; Fig. 3). There was no significant number of undifferentiated sperm cells for the sample of *X. muelleri*.

### Sperm Motility

Motility of sperm is important to successful fertilization in amphibian species. *Xenopus*, like many amphibian species, have external fertilization and sperm become motile and swim to fertilize eggs only after entering a lower osmolality environment [33]. Hybrids produce a lower abundance of mature sperm and while sterility may be explained in part by this lower abundance, sterility could be complete if sperm in hybrids failed to become motile. We tested the null hypothesis that sperm motility was the same in hybrids compared to the two parental species by counting the number of motile and immotile sperm following activation.

**Figure 4:**
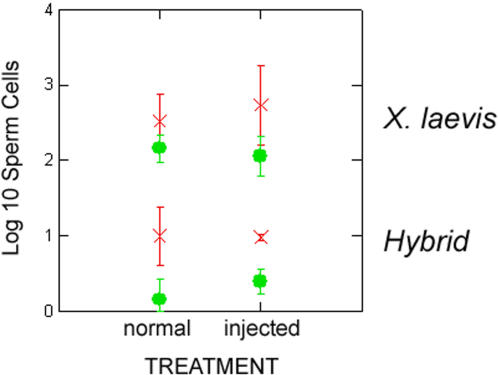
Numbers of motile (green circles) and immotile (red x's) sperm in *Xenopus laevis* and hybrids in normal/uninjected compared to hCG injected males. Error bars represent±1 standard error.

There were about 70 times more motile sperm in *X. laevis* compared to hybrids (*F*_1,5_ = 188.3; *P* = 0.000) and there was no effect of hCG treatment on the number of motile sperm (*F*_1,5_ = 0.258; *P* = 0.633). Despite the large difference in number of motile sperm cells, proportions of motile to immotile sperm were the same between *X. laevis* and hybrids (*F*_1,5_ = 0.570; *P* = 0.484; Fig. 4). There was 12% motile and 88% immotile sperm out of a total of 238 sperm cells observed in *X. muelleri*. This was close to the mean percentage of sperm for *X. laevis* (Mean = 18% motile and Mean = 82% immotile).

### Sperm Morphology

Abnormal sperm morphology is frequently associated with infertility [34] and misshapen or abnormal sperm morphology in hybrid males would suggest an explanation for sterility in hybrids. We measured the area of sperm cells as a proxy for size and tested the null hypothesis that the size of mature sperm was the same in hybrids compared to the parental species.

**Figure 5:**
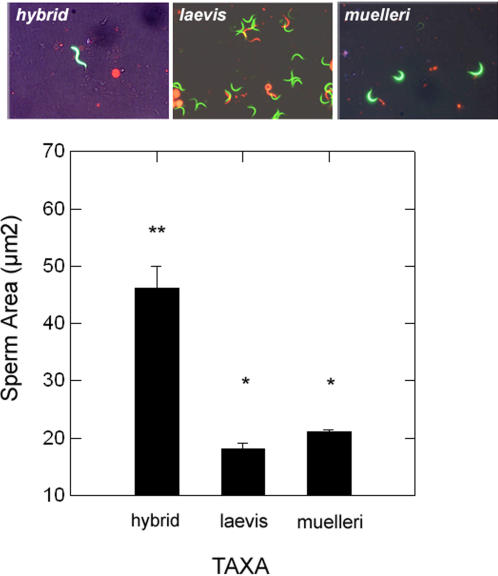
Comparison of sperm area (µm^2^) in *Xenopus laevis*, *X. muelleri*, and hybrids and representative brightfield images of sperm. Error bars represent±1 standard error and * denotes significance from a Bonferroni multiple comparison test. Hybrids have larger sperm compared to *X. laevis* and *X. muelleri* but sperm area does not differ between *X. laevis* and *X. muelleri*.

Hybrid sperm were dramatically larger compared to the sperm of *Xenopus laevis* and *X. muelleri* (*F*_1,51_ = 74.0; *P* = 0.000). Hybrid sperm were about 28 µm^2^ larger than *Xenopus laevis* (Bonferroni corrected *P* = 0.000) and 25 µm^2^ larger than *X. muelleri* sperm (Bonferroni corrected *P* = 0.000). The size of sperm did not differ between *X. laevis* and *X. muelleri* (Bonferroni corrected *P* = 0.513; Fig. 5).

### Gene Expression

Our analyses of sperm characteristics in hybrids compared to *X. laevis* and *X. muelleri* revealed several phenotypic differences that may contribute to the sterility pattern. We next analyzed the transcriptional pattern in hybrids and the two parental species to explore the proximate mechanism of gene expression and its contribution to the phenotype of hybrids. This approach allowed us to identify loci that may be involved in generating the sterility phenotype and simultaneously examine the broad pattern of gene expression in hybrids compared to the parental species.

**Figure 6:**
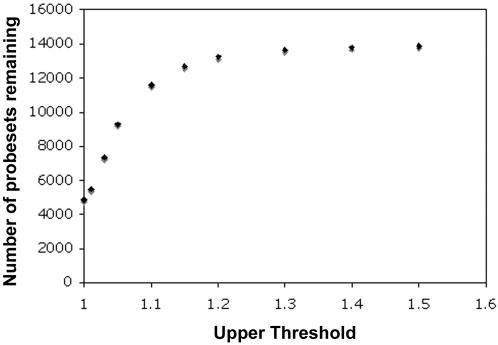
Number of probesets remaining at various thresholds from comparing the hybridization intensity of *Xenopus muelleri* vs. *X. laevis.*

We used Affymetrix *Xenopus laevis* Genome Arrays to generate a transcriptional profile of gene expression in testes of hybrids, *X. laevis*, and *X. muelleri*. The Affymetrix microarray is designed for *X. laevis* and it is widely accepted that hybridizing RNA from a heterospecific species to a microarray designed for a related species can have a dramatic impact on the signal recovered from microarrays [35]–[37]. To control for this effect we directly assayed sequence divergence by hybridizing genomic DNA from *Xenopus laevis* and *X. muelleri* each separately to the Affymetrix *Xenopus laevis* GeneChip® Genome Array. By taking the ratio of hybridization intensity of *X. muelleri*/*X. laevis* for each probe on the array we then screened out probes that did not hybridize properly due to sequence divergence in *X. muelleri*. To be conservative we set the lower ratio of hybridization intensity to 0.99 and explored variation in the number of probes eliminated at a variety of upper thresholds with the idea that higher intensity for *X. muelleri* is not as damaging compared to probes that have a hybridization signal lower for *X. muelleri* when compared to *X. laevis* (Fig. 6). In examining the threshold variation on the number of probesets remaining, we chose to analyze the datasets generated at the 1.01 and 1.10 ratio level. Both datasets provided a similar general pattern but the effect was more prominent for the less stringent threshold. We report here the results generated using an upper threshold of 1.10 in subsequent analyses of gene expression which resulted in the removal of 226,841 individual probes and provided 11,485 probesets for further analysis. We tested the null hypothesis that the expression level for each of the 11,485 probesets was the same in three separate contrasts (hybrids vs. *X. laevis*; hybrids vs. *X. muelleri*; and *X. laevis* vs. *X. muelleri*).

**Figure 7:**
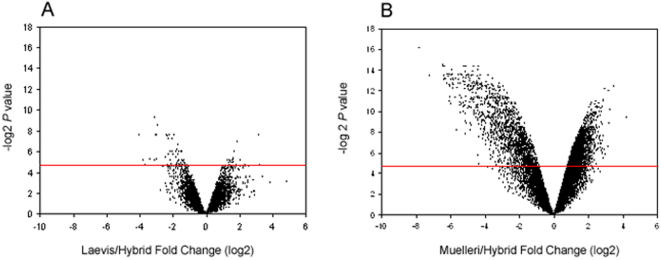
Volcano plots from FDR corrected *t*-tests of statistical significance (vertical axis) against magnitude of expression change (horizontal axis), where each point corresponds to a gene/transcript. Expression change (fold-change) is defined as a log_2_-transformed ratio of mean nonhybrid to mean hybrid expression level. (A) *Xenopus laevis* vs. Hybrids; (B) *Xenpous muelleri* vs. Hybrids. The red line denotes FDR adjusted alpha 0.05. The horizontal deviation from 0 towards the right or left reflects hybrid underexpression or overexpression, respectively.

Microarray analysis revealed an asymmetrical pattern of gene misexpression between hybrids and the two parental species. Only 1.2% of genes (142/11,485) in our sample of the transcriptome were misexpressed between *Xenopus laevis* and hybrids whereas about 35% of genes (3,995/11,485) were misexpressed between *X. muelleri* and hybrids (Fig. 7). There were more genes upregulated in hybrids relative to *X. laevis* (92 vs. 50; *G* = 12.61; df = 1; *P*<0.001) but there were more genes upregulated in *X. muelleri* compared to hybrids (2,236 vs. 1759; *G* = 57.1; df = 1; *P*<0.001). Complete results for each of the three main contrasts can be found in Tables S1, S2 and S3. The top thirty most misexpressed transcripts in the two contrast tests were mainly dominated by EST sequences with little functional information available but our analysis of gene expression in the testis would suggest that these EST sequences have testis related function. Among the annotated transcripts, many are known to have functions in the process of spermatogenesis, spermiogenesis, or testis related functions or are involved in regulating polymerase II transcription (Table S4, S5, S6 and S7). There were 56 transcripts recovered as differentially expressed in both contrasts (Table 1) and these transcripts may have a more crucial role in the sterility pattern in hybrid males of *Xenopus*.

**Table 1:** Candidate loci (56 total) common to both contrasts (*X. laevis* vs hybrids; *X. muelleri* vs. hybrids) divided according to corresponding expression behavior (hybrid>(*laevis = muelleri*); hybrid<(*laevis* = *muelleri*); *laevis*>hybrid>*muelleri*; *laevis*<hybrid<*muelleri*).

				*X. laevis* vs. Hybrid Contrast				*X. muelleri* vs. Hybrid Contrast			
Probeset ID	Target Gene	Gene Symbol	Description/Molecular Function	Mean Laev.	Mean Hybrid	L-H	*P* Value	Mean Muell.	Mean Hybrid	M-H	*P* Value
Xl.25297.1.S1_at	ESTs			4.007	6.433	−2.426	0.0234	3.367	5.978	−2.611	0.0061
Xl.12561.1.S1_at	XMeis1-3	LOC398167	Homeodomain transcription factor	5.561	7.764	−2.202	0.0405	5.194	7.367	−2.173	0.0208
Xl.24218.1.S1_at	Cnef01			8.974	11.080	−2.105	0.0468	9.237	10.873	−1.636	0.0091
Xl.8717.2.A1_at	CD81 antigen	cd81-b	Target of antiproliferative antibody	7.013	9.094	−2.080	0.0348	7.432	8.765	−1.333	0.0248
Xl.18983.1.S1_at	ESTs	MGC82221	Zinc and metal ion binding and oxidoreductase activity	6.685	8.431	−1.747	0.0405	5.463	8.068	−2.606	0.0015
Xl.10985.1.A1_at	ESTs	MGC82900		7.799	9.232	−1.433	0.0387	7.876	8.936	−1.060	0.0254

				*X. laevis* vs. Hybrid Contrast				*X. muelleri* vs. Hybrid Contrast			
Probeset ID	Target Gene	Gene Symbol	Description/Molecular Function	Mean Laev.	Mean Hybrid	L–H	*P* Value	Mean Muell.	Mean Hybrid	M-H	*P* Value
Xl.22458.1.A1_at	ESTs			9.905	6.673	3.232	0.0393	10.466	6.230	4.236	0.0015
Xl.13889.1.A1_at	ESTs			7.259	5.332	1.926	0.0084	6.190	4.888	1.302	0.0332
Xl.19899.1.A1_at	ESTs		Similar to bromodomain, 2B (Rattus norvegicus)	5.026	3.844	1.182	0.0484	6.275	3.514	2.760	0.0008

				*X. laevis* vs. Hybrid Contrast				*X. muelleri* vs. Hybrid Contrast			
Probeset ID	Target Gene	Gene Symbol	Description/Molecular Function	Mean Laev.	Mean Hybrid	L-H	*P* Value	Mean Muell.	Mean Hybrid	M-H	*P* Value
Xl.5076.1.S1_at	ESTs	LOC495423	Nucleotide and ATP binding; kinase activity	7.099	4.697	2.403	0.0390	3.285	4.298	−1.012	0.0449
Xl.10826.1.A1_at	ESTs	MGC82938	Moderately similar 5-AMP-activated protein kinase (Homo sapiens)	8.377	6.514	1.863	0.0405	3.945	6.082	−2.136	0.0079
Xl.23530.1.S1_at	MGC52668		Trafficking protein particle complex 6A (Xenopus tropicalis)	8.046	6.326	1.720	0.0151	3.757	5.879	−2.121	0.0099
Xl.18999.1.A1_at	RALDH2	ALDH1A2	Oxidoreductase activity	8.074	6.400	1.674	0.0248	4.680	6.327	−1.647	0.0049
Xl.6988.1.A1_at	ESTs	MGC82200	Protein binding and transporter activity	9.027	7.370	1.658	0.0320	4.850	6.953	−2.103	0.0167
Xl.15612.1.A1_at	ESTs			7.059	5.423	1.636	0.0408	3.847	4.949	−1.102	0.0402
Xl.5907.1.A1_at	ESTs			9.354	7.807	1.547	0.0454	4.021	7.413	−3.392	0.0033
Xl.7749.1.A1_at	ESTs			8.501	7.201	1.300	0.0269	4.252	6.777	−2.525	0.0168
Xl.20487.1.A1_at	ESTs			8.135	6.876	1.259	0.0484	2.972	6.434	−3.462	0.0012
Xl.12917.1.A1_at	ESTs			8.494	7.352	1.143	0.0405	4.940	6.935	−1.995	0.0302
Xl.4873.1.S1_at	nucleostemin	wu:fc55d07	Protein, nucleotide, and GTP binding; regulation of cell cycle	11.053	10.008	1.045	0.0408	6.970	9.719	−2.749	0.0004
Xl.25940.1.S1_at	ESTs	MGC82032	Adenosine kinase activity; purine ribonucleoside salvage	8.972	7.948	1.024	0.0454	3.749	7.561	−3.812	0.0001
Xl.5178.2.A1_at	actin related ei	arpc1a	Actin binding and cellular morph.	10.780	9.786	0.993	0.0471	4.319	9.489	−5.170	0.0001

				*X. laevis* vs. hybrid contrast				*X. muelleri* vs. hybrid contrast			
Probeset ID	Target Gene	Gene Symbol	Description/Molecular Function	Mean Laev.	Mean Hybrid	L-H	*P* Value	Mean Muell.	Mean Hybrid	M-H	*P* Value
Xl.2789.1.A1_at	ESTs		Similar to putative lymphocyte G0/G1 switch protein 2 (Gallus gallus)	4.109	6.347	−2.237	0.0470	8.615	5.900	2.716	0.0085
Xl.8916.1.S1_at	ESTs			3.883	7.225	−3.342	0.0256	9.004	6.801	2.202	0.0028
Xl.3481.1.S1_at	ESTs		Highly similar to ubiquitin-like 5 (Homo sapiens) (H.sapiens)	3.683	6.290	−2.607	0.0418	7.988	5.853	2.135	0.0286
Xl.6034.1.A1_at	ESTs	MGC82505	Lipid binding	6.973	8.720	−1.747	0.0344	10.434	8.373	2.061	0.0054
Xl.19741.1.S1_at	ESTs	LOC495153		5.052	7.137	−2.085	0.0281	8.731	6.708	2.023	0.0315
Xl.20475.1.S1_at	ESTs			3.535	5.677	−2.142	0.0405	7.117	5.234	1.884	0.0221
Xl.21956.1.S1_at	ESTs	MGC53461		6.978	10.026	−3.048	0.0016	11.611	9.739	1.872	0.0015
Xl.12866.1.S1_at	ESTs	G6pc	Glucose-6-phosphotase activity	5.006	6.297	−1.291	0.0384	7.640	5.833	1.807	0.0037
Xl.16399.1.S1_at	MGC53769	dkfzp434h2226		9.286	10.684	−1.398	0.0405	12.243	10.438	1.805	0.0035
Xl.11954.1.S1_at	PAK5	PAK5 protein	Protein amino acid phosphorylation	6.897	9.269	−2.373	0.0053	10.759	8.962	1.797	0.0036
Xl.25810.1.A1_at	ESTs	LOC443658	Protein, zinc, and metal ion binding	6.649	8.321	−1.672	0.0188	9.755	7.979	1.776	0.0040
Xl.23573.1.S1_at	TDG		DNA repair; hydrolase activity, acting on glycosyl bonds	3.862	6.142	−2.280	0.0399	7.423	5.679	1.744	0.0443
Xl.16776.3.A1_at	ESTs	MGC68565	Protein binding	6.005	8.865	−2.860	0.0027	10.213	8.526	1.688	0.0108
Xl.252.1.S1_s_at	erg	erg-A; erg-B	Transcription factor activity; sequence-specific DNA binding	3.272	5.570	−2.298	0.0408	6.808	5.128	1.680	0.0220
Xl.16427.1.A1_at	ESTs		Similar to guanine nucleotide exchange factor (1B384) (Gallus gallus)	6.335	7.851	−1.516	0.0405	9.109	7.458	1.651	0.0111
Xl.24133.1.S1_at	eIF4G protein	NAT1	RNA binding; translation initiation factor activity	9.142	10.187	−1.044	0.0471	11.538	9.909	1.630	0.0055
Xl.14571.2.S1_at	ESTs	LOC495071	Protein, zinc, and metal ion binding	6.680	8.146	−1.466	0.0344	9.426	7.812	1.615	0.0116
Xl.22032.1.A1_at	ESTs			5.386	6.469	−1.083	0.0455	7.758	6.294	1.464	0.0047
Xl.9401.1.A1_at	ESTs			7.766	9.403	−1.637	0.0097	10.541	9.118	1.423	0.0048
Xl.3958.1.A1_at	ESTs			7.515	10.445	−2.929	0.0053	11.593	10.186	1.407	0.0159
Xl.25538.1.A1_at	ESTs			8.281	9.853	−1.571	0.0408	10.969	9.570	1.399	0.0213
Xl.6844.1.A1_at	ESTs	MGC83883	Kinase activity	5.987	7.841	−1.854	0.0405	8.816	7.448	1.368	0.0236
Xl.26410.1.A1_at	ESTs	MGC84299	Syntaxin binding	5.946	7.373	−1.426	0.0154	8.316	6.974	1.342	0.0059
Xl.8719.1.S1_at	ESTs	dynein light chain 2	Microtubule motor activity	7.985	10.176	−2.191	0.0053	11.184	9.898	1.285	0.0113
Xl.525.1.S1_at	forkhead box D3	foxd3-A	Transcription factor activity; regulation of transcription	4.656	5.756	−1.099	0.0471	6.677	5.391	1.285	0.0079
Xl.2331.1.A1_at	ESTs			7.549	8.748	−1.199	0.0454	9.672	8.402	1.269	0.0078
Xl.11050.2.A1_at	ESTs	MGC80999	Weakly similar to hypothetical protein FLJ20125 (Homo sapiens)	7.291	9.389	−2.098	0.0152	10.294	9.077	1.217	0.0108
Xl.26364.1.A1_at	ESTs	MGC84386		6.704	8.026	−1.322	0.0323	8.802	7.642	1.160	0.0225
Xl.24104.1.A1_at	ESTs			6.105	7.375	−1.270	0.0405	8.118	6.961	1.157	0.0106
Xl.10225.1.A1_at	ESTs		Similar to hypothetical protein FLJ14490 (Pan troglodytes)	9.379	11.188	−1.809	0.0160	12.105	10.989	1.116	0.0242
Xl.3975.1.A1_at	ESTs	LOC495973		7.444	9.321	−1.877	0.0053	10.027	9.016	1.011	0.0189
Xl.1555.1.A1_at	ESTs			7.694	9.197	−1.503	0.0160	9.869	8.870	0.999	0.0201
Xl.2895.1.A1_at	ESTs			4.752	5.912	−1.160	0.0393	6.464	5.501	0.963	0.0289
Xl.449.1.S1_at	hermes	LOC398092	Nucleotide binding; nucleic acid binding; RNA binding	6.534	8.252	−1.718	0.0137	8.522	7.646	0.876	0.0367

Expression values are in log2 scale. *P* values are FDR adjusted values.

There was also a dramatic difference in expression between the two parental species. About 60% of genes were differentially expressed between *X. laevis* and *X. muelleri* (6956/11485). Of these differentially expressed transcripts, about 40% (2824/6956) were upregulated in *X. laevis* relative to *X. muelleri* and 60% (4132/6956) were upregulated in *X. muelleri* relative to *X. laevis*.

Comparing the overlap in genes misexpressed in individual hybrid contrasts to the three classes of expression behavior between *X. laevis* and *X. muelleri* (*X. laevis*>*X. muelleri; X. laevis*<*X. muelleri*; *X. laevis = X. muelleri*) revealed that in general hybrids have an intermediate level of expression compared to the two parental species (Table 2). For example, of the 92 genes upregulated in hybrids relative to *X. laevis*; 92% were also upregulated in *X. muelleri* relative to *X. laevis*. Similarly, of the 1,759 genes that were upregulated in hybrids relative to *X. muelleri*, 92% were upregulated in *X. laevis* compared to *X. muelleri*. These results suggest a general pattern of intermediate expression in hybrids and are consistent with a semi-dominant model of expression difference even despite the strong asymmetrical pattern of misexpression in hybrids compared to the two parental species.

**Table 2:** Comparison in the overlap of transcripts recovered as differentially expressed from the two contrasts with hybrids (*Xenopus laevis* vs. hybrids and *X. muelleri* vs. hybrids) and the interspecies contrast (*Xenopus laevis* vs. *X. muelleri*).

		*X. laevis* vs. Hybrids		*X. muelleri* vs. Hybrids	
		L<H	L>H	M<H	M>H
*X. laeivs* vs. *X. muelleri*	L>M	0	41	1626	1
	L<M	85	1	1	2029
	L = M	7	8	132	206
	Total	92	50	1759	2236

The congruence between patterns of expression behavior in hybrids compared to the interspecies comparison suggests a model of semidominance where hybrids have an intermediate level of expression compared to the two parental species.

We further asked whether the observed expression pattern is unbiased by sequence divergence still unaccounted for by the masking procedure or distorted by the procedure itself. To address this question, we used genomic sequence information available for *Xenopus tropicalis* (JGI, v3.0) as an outgroup to help identify highly conserved sequences among distantly related species. *X. tropicalis* belongs to a clade (*Silurana*) that have diverged from *X. laevis* and *X. muelleri* for more than 70 million years, compared to a >20-million year divergence between *X. laevis* and *X. muelleri* [38], [39]. We blasted (BLASTN 2.2.12 [40]) all 495,232 *X. laevis* probe sequences provided by Affymetrix against the *X. tropicalis* genome and selected 20 probesets with the lowest E-values (10^−6^ to 0.04 averaged across the 32 probes per probeset) corresponding to the most conserved sequences (100-97.2% mean identity) between *X. laevis* and *X. tropicalis* (Table S8). As these two species are more distantly related than *X. laevis* and *X. muelleri* are to each other, it is reasonable to assume that these sequences will tend to be conserved in *X. muelleri* as well (no *X. muelleri* genome information is available), and thus their expression pattern will not be confounded by sequence divergence. We also reasoned that if the masking procedure was valid and unbiased, the expression patterns from the mask and the most conserved probesets would be similar. Indeed, the mean differences between normalized expression levels from these two methods were almost identical: 0.503 and 0.427 for the *X. laevis*-hybrids contrast and 1.040 and 1.091 for the *X. muelleri*-hybrids contrast (Kolmogorov-Smirnov tests, *P*>0.05). This approximately twofold expression difference between the *X. laevis*-hybrid and the *X. muelleri*-hybrid comparisons from the conserved probesets was significant (t-test, *P* = 0.029), confirming the asymmetric pattern of expression differences. This asymmetry persisted across all masking criteria, in the absence of the mask (*X. laevis* vs. hybrids: 180/15491 = 1.2%, *X. muelleri* vs. hybrids: 9345/15491 = 60.3%), and was robust to changes in the method of normalization (RMA vs. MAS 5.0 scaling – results not shown), providing additional support for the expression pattern.

## Discussion

Our analysis of sperm abundance, motility, and morphology in hybrids suggests that the process and control of spermatogenesis is severely perturbed in hybrids. Hybrids have about 40 times less total sperm compared to parental species. Mature sperm in hybrids are capable of motility and the highly reduced abundance of sperm suggests that these numbers may be insufficient to fertilize eggs *in vivo*. Additionally, differential live/dead staining of sperm cells in hybrids showed that the majority of cells consisted of undifferentiated sperm suggesting an overall pattern of arrested development of spermatogenesis and/or spermiogenesis. Cells that have the characteristic shape of sperm in hybrids are larger compared to *X. laevis* and *X. muelleri* raising the possibility that these sperm cells have abnormal numbers of chromosomes. Kobel (1996) [18] suggested that sperm cells of hybrids were aneuploid and our results may be consistent with these observations. Future work focusing on chromosome content of hybrid sperm would aid in determining the end result of meiosis in hybrids.

Sperm production in hybrids did not respond to hCG hormone treatment whereas the proportion of dead cells increased dramatically in *X. laevis*. The dosage of hCG used in the experiment is typical for stimulating reproduction in *Xenopus* but our results suggest the possibility that the dose may be too high. hCG triggers the production of LH and FSH which are involved in the differentiation of sperm [31], [32]. Overstimulation by excessive hCG could result in sperm death and may explain the increased numbers of dead sperm in *X. laevis* but why hybrids fail to respond to hCG treatment remains an intriguing question. If receptors for hCG are more sensitive in hybrids, an abnormally high dose of hCG may cause these receptors to become inactivated leading to a lack of response by the process of spermatogenesis. Alternatively, hormone receptors in hybrids may fail to function properly due to the hybrid genetic background which could lead to a lack of response to hCG treatment. This later hypothesis would implicate a possible mechanism for the overall different pattern of spermatogenesis observed in hybrids. Spermatogenesis, a process tightly controlled by hormonal interactions with the hypothalamic-pituitary-gonadal axis [30], may be misregulated due to improper interactions with hormones and receptors. This hypothesis has received no investigation in studies of reproductive isolation despite considerable attention directed to the process of spermatogenesis as an explanation for Haldane's rule [41], [42]. The lack of response to hCG in hybrids suggests that hormonal regulation of spermatogenesis may contribute to reproductive isolation in hybrids and support for this hypothesis could be generated by assaying receptor function in hybrids compared to parental species.

The genes misexpressed in hybrids may offer clues to the loci involved in the sterility phenotype characteristic of hybrid males and therefore potential loci that contribute to reproductive isolation between *X. laevis* and *X. muelleri*. Many of the most misexpressed loci consist of EST targets and therefore currently there is little functional information available. One EST of interest though has been identified to be the transcription factor TFIIE complex which is a transcription factor important to the function of RNA polymerase II transcription. This EST was downregulated 4 times in hybrids relative to *X. laevis* suggesting that Pol II transcription may be impacted by the lack of TFIIE in hybrids. Interestingly, this gene was not found to be misexpressed compared to *X. muelleri*.

Many interesting genes related to spermatogenesis in other organisms appear in the candidate gene lists. For example, type 2 retinaldehyde dehydrogenase (*RALDH2*) was found to be 3.2 times lower in hybrids compared to *X. laevis*. This gene catalyzes the important developmental modulator retinoic acid and is exclusively expressed in mouse testis [43]. Caesin kinase I (*CKIe*), a gene involved in protein amino acid phosphoryalation through the utilization of ATP but not GTP was downregulated 4.3 times in hybrids relative to *X. laevis* and a unique form of *CKle* is expressed in rat testis [44].

The most misexpressed gene in hybrids relative to *X. laevis* was neuropeptide Y (*NPY*). *NPY* is one of the most abundant and widespread nueropeptides in mammals and has been suggested to play a role in controlling reproductive function. In particular, *NPY* potentiates the release of LH and FSH in response to GnRH [45], [46] and is modulated by testosterone. *NPY* is predominantly synthesized in the hypothalamic arcuate nucleus but also expressed in the testes (mostly in Leydig cells) of mouse and rat [47], [48]. Importantly, there is a strong association between testicular development and the expression of *NPY* [48]. *NPY* was upregulated 16 times in hybrids relative to *X. laevis* and also was overexpressed in *X. muelleri* relative to *X. laevis*. Other upregulated genes in hybrids relative to *X. laevis* and that have documented expression patterns in the testes include *PAK5* (p21 activated kinase 2 plays a role in Sertoli-germ cell anchoring dynamics of rats; [49] and *dynll2* (dynein light chain-1 was highly expressed in mouse and rat testis; [50]).

Examining genes misexpressed in hybrids compared to *X. muelleri* reveals that there are also many interesting genes related to the function of the testis in other organisms. *Occludin* which was upregulated about 9 times more in *X. muelleri* compared to hybrids has an important role in the formation of tight junctions surrounding Sertoli cells and seminiferous tubules in mammals [51] and salamanders [52]. *Goosecoid* is an important transcription factor during early development of *Xenopus* and was found to be about 8 times overexpressed in *X. muelleri* relative to hybrids. A related gene (*goosecoid-like*) is expressed primarily in the brain and primoridial germ cells in mouse [53]. *B1*, a *X. borealis* specific gene included on the *X. laevis* microarray, was upregulated about 7 times in *X. muelleri* compared to hybrids and plays an important role in the assembly of TFIIIA and expression of 5S RNA by RNA polymerase III [54]. Two tubulin related genes (similar to *alpha-tubulin* and *beta-tubulin*) were overexpressed 140 and 85 times respectively in hybrids relative to *X. muelleri*. Beta tubulin 2 has a testis restricted expression profile in *Aedes aegypti* and *Drosophila* [55], [56]. Another gene of interest is cyclin B2, upregulated 70 times in hybrids compared to *X. muelleri*. Cyclins have a demonstrated role in spermatogenesis in the eel *Anguilla japonica* [57] and mouse [58] and play a role in cell cycle regulation particularly in response to cancerous aberrations in germ cells [59]. Ferritin was found to be upregulated 68 times in hybrids compared to *X. muelleri*. Recently mitochondrial ferritin has been discovered to have a testis specific expression profile in mammals and flies [60] leading to the possibility that we recovered a signal of this ferritin product in *Xenopus*. Finally, HMG box protein, a transcriptional repressor that was upregulated about 62 times in hybrids relative to *X. muelleri*, has been found to be expressed strongly in mouse testis [61].

The gene expression data suggest two major patterns for expression behavior in hybrids relative to the two parental species. First, for misexpressed genes in hybrids the overwhelming preponderance of misexpressed genes follow a semi-dominant model of expression behavior because hybrid expression is intermediate and/or additive compared to expression differences between the two species (Table 2). Hybrids of these two species of *Xenopus* then appear to follow a very different overall pattern of misexpression compared to hybrids in other organisms. For example, hybrids of *Drosophila* have distinctly nonadditive expression behavior because hybrids have more misexpression compared to the genes misexpressed between the two parental species [3]–[4]. The same can be said for hybrids of maize [62], *Arabidopsis* [8], [63]–[65], and *Senecio* [7]. In the case of *Xenopus*, more genes are misexpressed between species than for hybrids compared to each parental species.

The second major pattern observed from the microarray results is that there was a difference in the expression pattern of hybrids compared to the two parental species. Surprisingly, a relatively few number of genes were misexpressed between *X. laevis* and hybrids (142/11,485) while a substantial number of genes were misexpressed between *X. muelleri* and hybrids (3995/11485). In effect, adult testes of hybrids have an expression pattern very similar to *X. laevis* but substantially different compared to *X. muelleri*. These results suggest that most genes in the hybrid genetic background follow a dominant pattern of expression because the overall level of expression in hybrids is equal to that of *X. laevis* only. This pattern of expression in hybrid testes differs from analyses of proteins in hybrids of *X. laevis* and *X. borealis* (a related species to *X. muelleri*). Both species specific proteins of *X. laevis* and *X. borealis* are expressed in hybrid ovary [66] and species-specific copies of several allozyme loci appear to both be expressed in various developmental stages of tadpoles, adult heart, and adult liver [67]. However, LDH isozyme in early embryos resembled that of the maternal species (*X. laevis victorianus*) and this persisted through metamorphosis [68]. These studies suggest that the allelic dominance observed in the expression profile of hybrid testes is not distributed in other tissue types but rather may be confined to the testes.

One interpretation of these results is that the collective molecular processes that occur in adult frog testes (i.e. spermio- and spermatogenesis) are under the influence of massive genomic imprinting and specifically, the maternal but not the paternal copies of genes are expressed. This would provide an explanation for the similarity of hybrid expression profiles to *X. laevis* and the dissimilarity compared to *X. muelleri* because the mother of these hybrids is *X. laevis* and not *X. muelleri*. No evidence for imprinting has been found for *Xenopus* but this hypothesis would require that imprinting may occur in *Xenopus* and that maternal imprinting is the controller of the expression pattern in whole frog testes [69]. One fruitful avenue to explore this hypothesis would be to analyze the expression profiles from testes of reciprocal cross hybrids (*X. muelleri* x *X. laevis*). If the expression profile becomes more similar to *X. muelleri* then this would be evidence of strong maternal effects, possible related to genomic imprinting, controlling the expression pattern in frog testes.

An alternative mechanism that may explain the dissimilar expression profiles is that in hybrids the paternal copy of chromosomes is eliminated or quiescent at the expression level. Hybrids then would express only the maternally contributed *X. laevis* portion of the genome but this would require that the maternally contributed portion be upregulated two fold to match the expression profile of *X. laevis*. The mechanisms involved here are difficult to explain but one possibility may be that paralogous gene copies, normally repressed, become expressed thereby providing expressed products to match that of *X. laevis*. Genomic elimination of paternal copies does occur in insects and frogs [70]–[74]. For example in the treefrog, gene silencing and monoallelic dominance of one genome occurs in crosses of tetraploid *Hyla versicolor* and diploid *H. arborea* [72]. In F1 triploid hybrids, several allozyme loci had unexpected frequencies suggesting that in some cases the paternal gene was silenced (i.e. *Mpi-2*) or that the maternal allele was silenced and the single paternal allele had double expression (i.e. *Got-1*). The implication here is that the foreign copy can be recognized and eliminated, and given the polyploid nature of *Xenopus* a similar mechanism may be operating. Indeed, amphibians have remarkably examples of genomic exclusion. One well documented example consists of the hybridogenetic system of the *Rana esculenta* complex in Europe where the entire paternal genome is eliminated during oogenesis [75].

Bringing together two divergent genomes into a common genetic background can induce a variety of genomic changes including genome instability, changes in chromatin, and transcriptome shock [7], [76]–[79]. In particular, gene silencing appears to be one major result of bringing together two divergent genomes into a common genomic environment via hybridization. For example, interesting patterns regarding rRNA gene expression have been established in interspecific hybrids of *Xenopus*. Both *Xenopus laevis* and *X. borealis* (a species closely related to *X. muelleri*) display two nucleoli per cell during embryogenesis. Hybrids between these two species have one nucleolus in the majority of cells suggesting that the nucleolar organizer is inactivated due to genomic incompatibility between the two species [80]. More specifically, in hybrids between *Xenopus laevis* and *X. borealis* (in the cited papers mistakenly referred to as *X. muelleri*; [81], the rRNA of *X. laevis* is preferentially transcribed and *X. borealis* is repressed [82]. Repression of *X. borealis* rRNA is basically complete until the swimming tadpole stage during which a low level of *X. borealis* rRNA is detectable. In adults, the level of expression of the *X. borealis* copy ranges from zero to significant amounts indicating the presence of much variation between individual hybrids. This phenomenon, first called “differential amphiplasty” and currently termed “nucleolar dominance”, is an epigenetic phenomena that is controlled at the level of transcription and occurs in a wide variety of interspecific hybrids including the plant taxa *Crepis* [83], [84], *Salix* [85], *Ribes* [86], [87], *Solanum* [88], *Hordeum* [89]–[92], *Triticum* [93]–[96], *Agropyron* [97], *Brassica* [98], [99], and *Arabidopsis* [100] as well as hybrids of *Drosophila* [101], [102], and mammal somatic cell hybrids of mouse and human [103]–[109]. Nucleolar dominance operates on tens of millions of base pairs of chromosomal DNA and this large scale inactivation mechanism is only rivaled by X chromosome inactivation in mammalian cells [110].

Can the mechanisms of nucleolar dominance, a pattern specifically related to the RNA polymerase I transcription machinery of rRNA and not with RNA polymerase II transcription of protein coding genes provide explanations for the asymmetrical transcriptomic expression pattern observed in hybrids of *Xenopus*? The mechanisms that discriminate maternal or paternal rRNA genes remain unclear but three hypotheses have been proposed to account for the differential silencing of species specific alleles: the species-specific transcription factor hypothesis, the enhancer-imbalance hypothesis, and the chromatin imprinting hypothesis [111]–[113].

### Species-Specific Transcription Factor Hypothesis

The DNA sequences coding for ribosomal proteins (18S, 5.8S, and 25/26S) are highly conserved but intergenic rDNA sequences are highly divergent amongst eukaryotes [112]–[115]. According to the species-specific transcription factor hypothesis, because of this divergent evolution rRNA gene transcription can occur only if co-evolved species-specific transcription factors bind to corresponding regulatory sequences. When genomes from two different species are brought together, there is a failure of rDNA transcription because of a lack of appropriate specific transcription factors. Cell-free transcription systems of mouse-human hybrid cell lines revealed that the promoter for mouse or the promoter for human rRNA would not function in a cell-free extract made from another species [116]–[119]. In plants, the tomato rRNA gene promoter does not work when transfected into *Arabidopsis* protoplasts and the tobacco rRNA gene promoter does not work when placed into a bean cell-free extract [120], [121]. These findings suggest that transcription factors had co-evolved with their corresponding regulatory DNA sequences because transcription function was erased when forced into a heterospecific environment.

One prediction of this co-evolution model is that the amount of sequence divergence would be inversely related to how effective the mechanisms of the species-specific transcription hypothesis operate and this appears to be the case. When more closely related species are brought together into a common genomic environment, rRNA gene promoters are fully functional. For example, in *Brassica napus*, a hybrid derived from *B. rapa* and *B. oleracea*, only *B. rapa* rRNA is transcribed reflecting a pattern of nucleolar dominance but both rRNA genes were transcribed when transfected into protoplasts of the other species [122]. The same pattern occurs in *Arabidopsis suecica*, a hybrid of *A. thaliana* and *A. arenosa*; *A. thaliana* and *A. arenosa* rRNA genes are expressed in both species and transfected *A. thaliana* rRNA is active when introduced into the hybrid [100]. These congeneric interspecific hybrids are more closely related compared to the previous examples and here species specific transcription factors work fine in a heterospecific background. In the case of *Xenopus*, it is possible at least for some loci that transcription factors may fail to bind to their corresponding regulatory targets causing a silencing of underdominant genes because the two species examined in this study are substantially divergent. The estimated divergence time for the last common ancestor of *X. laevis* and *X. muelleri* is 21–35 mya [38], [39], [123]. However, this mechanism could operate on a genome wide scale in adult testes only if a key transcription factor, or factors, derived from maternally contributed *X. laevis* genes and involved in the regulation of *X. muelleri* chromatin was sufficiently divergent to misregulate the proper expression of *X. muelleri* alleles. Allele-specific imprinting at the level of chromatin has been recently reported in *Arabidopsis* [124] and in the case of *Xenopus* evidence for this hypothesis would be obtained by identifying chromatin remolding factors that operate from the maternal genome to regulate paternal copy chromatin and/or paternal copy transcription.

### Enhancer Imbalance Hypothesis

The enhancer imbalance hypothesis proposes that differences in the amount of intergenic sequences provide a competitive environment for the transcription of rRNA genes. If particular intergenic sequences are more effective at controlling transcription then these sequences will outcompete sequences contributed by the other species creating an imbalance in the way that enhancers operate to sequester transcription factors. In *Xenopus*, the repetitive DNA elements located upstream of the rRNA promoter act as orientation and position independent enhancers of transcription [125]–[128]. In *X. laevis*, each intergenic spacer contains a 60/81 bp repeat with a 42 bp core element that shares 90% sequence similarity with the gene promoter. In *X. borealis*, complete 60/81 bp repeats are absent but the 42 bp core element is present suggesting that the 42 bp core element controls the enhancer effect [111], [129]–[131]. In hybrids, only the rRNA genes of *X. laevis* are transcribed [80], [81], [132] and this appears to be due to the more numerous enhancer elements located in *X. laevis* sequence compared to *X. borealis* which sequester a critical transcription factor necessary for transcription [126], [133], [134]. Co-injection of rRNA minigenes with complete spacers into oocytes of *X. laevis* and *X. borealis* showed that *X. laevis* minigenes were preferentially transcribed and this occurred even when injecting recombinant constructs that had spacer and promoter regions swapped [134]. These experiments support the notion that species specific differences in intergenic regions, and not gene promoters, are responsible for the failure of the *X. borealis* allele to be transcribed in hybrids.

The enhancer imbalance hypothesis stems directly from biochemical interactions of transcription factors and the enhancer elements that regulate gene expression and therefore follows models of *cis-trans* evolution that have recently been proposed [135], [136]. Differences in genomic content between species then may be consistent with this model because differing amounts of intergenic DNA and/or enhancers may cause problems with transcribing one allele from one of the two species. Examining published C-values for *Xenopus* (available on the Animal Genome Database-http://www.genomesize.com/), the genomic content of *Xenopus laevis* and *X. muelleri* differs by about 430 Mb (Mean *X. laevis* 3231 Mb; Mean *X. muelleri* 3660 Mb; *t* = −2.76; df = 1; *P* = 0.02). By combining these two genomes into the same genomic environment, different enhancer content resulting from differences in genome size, may contribute to the patterns observed in gene expression of *X. laevis*, *X. muelleri*, and their F1 hybrid.

### Chromatin Imprinting Hypothesis

DNA methylation and covalent modifications of histone tails within nucleosomes (acetylation, phosphorylation, sumoylation, ubiquination, and methylation) have an impact on chromatin structure, gene transcription, and epigenetic information [137]. Indeed modifications to chromatin are applied and removed in a highly specific fashion leading to the proposition of a histone code that is read by chromatin-associated factors [138]. The chromatin imprinting hypothesis proposes that changes in the regulation of chromatin state explain the dominance seen in the preferential transcription of rRNA genes [111]. DNA hypomethylation and histone hyperacetylation correlate with transcriptional activity and DNA hypermethylation and histone hypoacetylation correlate with transcriptional silencing and therefore allele specific regulation of chromatin state may render some gene copies to be transcribed while rendering others to be silent [139].

There is a striking correlation between genome size and the direction of silencing in interspecific hybrids. On average, the parental species with the smaller genome is always dominant whereas under-dominant loci came from the smaller genome of the two parental species [111]. The argument here is that larger genomes have more heterochromatin and when combined into the same genomic environment via hybridization this increased heterochromatin content changes the spatial structure of the genome within the nucleus leading to preferential silencing of one parental copy. In effect, the positional configuration of chromatin in the hybrid nucleus is imbalanced and the genome with the larger content will be subjected to heterochromatinization via chromatin remolding mechanisms [140]. As stated above, the genomic content differs between *X. laevis* and *X. muelleri* and the smaller of the two genomes (*X. laevis*) is the species for which hybrids are most similar at the expression level. Additionally, several genes involved in chromatin maintenance are misexpressed in hybrids compared to *X. muelleri* but not in hybrids compared to *X. laevis* (i.e. *Ube2e2* that has ubiquitin-protein ligase activity and SET which is involved in chromatin remolding).

Our analysis of the phenotype of hybrids revealed a dramatically lower abundance of sperm in hybrids, increased numbers of undifferentiated sperm cells, a lack of response to hCG treatment, larger sperm compared to both parental species and even despite these phenotypic abnormalities, the presence of a few motile sperm. Coupling these phenotypic data with microarray analyses provides loci that may lead to the sterile phenotypic condition of hybrids. These data are important for identifying genes germane to postzygotic reproductive isolation between species and we present the first lists of these loci for *Xenopus*. At the broad gene expression level, more genes are misexpressed between species compared to hybrids. This is different compared to gene expression analyses of hybrids in other organisms where in general more misexpression occurs in hybrids. Genes that are misexpressed in hybrids compared to each species follow a semi-dominant model of expression behavior because most genes have an intermediate level of expression in hybrids. Despite the semi-dominance of misexpressed genes, there was strong pattern of dominance for the *X. laevis* genome because hybrids had fewer genes misexpressed compared to *X. laevis* than compared to *X. muelleri*. This pattern implies a silencing mechanism for the paternally inherited *X. muelleri* alleles and has implications for the evolution of species differentiation at the expression level [141]. This silencing mechanism may be a natural phenomenon related to amphibian testis function or alternatively could be a genuine response to the hybrid genetic background. Most interesting is that this pattern of silencing, reminiscent of the widespread phenomenon of nucleloar dominance, has also been documented in *Arabidopsis* allotetraploid hybrids [65]. We propose three hypotheses derived from large scale silencing of *Xenopus* rRNA genes which could explain the species-level dominance pattern. By doing so we focus much needed attention on the molecular mechanisms that promote gene transcription and in particular how these mechanisms relate to the expression basis of reproductive isolation in *Xenopus*.

Future research will be required to address the intriguing question of why *Xenopus* do not conform to Haldane's rule, this longstanding generalization of evolutionary biology. There is a common misconception that Haldane's rule does not apply to taxa that lack heteromorphic sex chromosomes, which is an example of confusing the phenomenon (heterozygous sex is afflicted) with its cause (dominance patterns due to heteromorphic sex chromosomes). In fact, sterility patterns (but not inviability patterns) in various taxa lacking heteromorphic sex chromosomes conform well to Haldane's rule [142]–[144], and suggest a role of other evolutionary mechanisms, such as faster male evolution [42].

## Materials and Methods

### Experimental Crosses

Four biological replicate hybrid clutches were produced at The University of Texas at Arlington. The female parent for each of these crosses was *Xenopus l. laevis* and these originate from the Cape region of South Africa or from the laboratory of Jacques Robert at The University of Rochester. The male parents were *X. muelleri* and originate from Nkambeni area of Swaziland and were kindly provided by R. C. Tinsley. For the expression analysis, we deliberately used *X. laevis* from genetically diverse sources (Xenopus Express, J. Robert's Lab, and R. C. Tinsley's Lab) to increase intragroup heterogeneity resulting in the lower rate of the null hypothesis rejections and more conservative statistical inference.

Crosses were conducted in the following manner: each female was injected with 500 IU human chorionic gonadotropin (hCG; Choluron or Carolina Biological Supply) and placed into a container with 10 L of dechlorinated water. Males were injected with 200 IU hCG and then paired with individual females. Each hybrid clutch was raised to sexual maturity and clutches were maintained in 12L:12D photoperiod at 25°C air temperature. Animals were fed *Xenopus* pellets from Xenopus Express Inc. every two days.

### Sperm Assays

We quantified characteristics of the sperm in hybrids and the two parental species. From each of three biological replicate clutches we selected two sexually mature hybrids. Sexual maturity was determined by the presence of dark colored nuptial pads indicative of circulating androgens and characteristic of sexual maturity [145]. One hybrid was injected with 150 IU hCG and the other was not injected with hCG in order to test the effect of hCG treatment on sperm abundance. For *X. laevis*, we used F1 males from Xenopus Express and whose parents originate from the Cape of South Africa and/or the lab of J. Roberts at the University of Rochester and selected animals based on sexual maturity. Again, one of the two animals was injected with 150 IU hCG and then left overnight in 10 L of dechlorinated water. For *X. muelleri* we selected one F1 male *X. muelleri* that was produced at the University of Texas at Arlington and whose parents originate from the Nkambeni area of Swaziland. This male was injected with 150 IU hCG.

Males were killed by immersion in MS-222 and testes were harvested and placed in 500 µl of DeBoer's Solution (110 mM NaCl; 1.3 mM CaCl_2_; 1.3 mM KCl). Testes were homogenized using a handheld pestle to form a stock sperm solution. Ten µl of the stock sperm solution was aliquoted and further diluted 3∶1 with distilled water. The 3∶1 dilution ratio was found to provide the maximum number of motile sperm in *X. laevis* [33] and was used to assay for sperm motility. Motile sperm and nonmotile sperm were enumerated during a 2 minute interval. This procedure was repeated four subsequent times/individual to provide a metric of sperm motility. Two individuals per treatment (injected and uninjected) and per taxa (*X. laevis* and hybrids) were used for sperm motility assays.

We assayed the abundance of live and dead sperm cells using the LIVE/DEAD Sperm Viability Kit from Molecular Probes (Invitrogen, Inc.). The LIVE/DEAD Sperm Kit is designed specifically for assaying live and dead sperm cells. Five µl of a 50-fold dilution of the stock SYBR 14 dye and 5 µl of 2.4 mM propidium iodide were added to the stock sperm suspension followed by a 10 minute incubation at 36°C for each dye. Stained cells were collected on black polycarbonate filters (1.0 µm pore-size, GE Osmonics) and enumerated at a magnification of 165× using epifluorescence microscopy (Olympus BH-2). Briefly, 10 µl of stained sperm were added to 2 ml of particle-free distilled-water (0.2 µm pore-size filtered) contained in the filter tower of a 10- place filtration unit (Hoeffer). Following filtration, filters were placed on slides, a drop of immersion oil added to the surface, and a cover slip placed on top. Live and dead sperm cells were enumerated from fifteen separate, randomly-selected visual fields. Images were captured using a digital camera (Olympus DP70).

We measured the area of sperm acrosomes in square micrometers as a proxy for the size for spermatozoa using images captured from placing 10 ul of sperm stock solution onto a microscope slide. Thirteen sperm cells from hybrids, 21 sperm cells from *X. laevis*, and 20 sperm cells from *X. muelleri* were selected at random from the images and the area in square micrometers of each sperm cell was measured using ImageJ software (NIH).

We constructed general linear models in SYSTAT v. 8.0 (SAS, 1998) to test the null hypothesis that there was no difference in the total number of sperm cells, the proportion of live, dead, and undifferentiated sperm cells, and the proportion of motile and nonmotile sperm between taxa and between the hCG treatment. An ANOVA was used to test the hypothesis that there was no difference in the size of spermatozoa between treatments.

### Microarray Experiments

RNA was extracted from adult testis in *Xenopus laevis* (n = 4), hybrids of *X. laevis* x. *X. muelleri* (n = 4) and *X. muelleri* (n = 3). Adults were euthanized with MS-222 and testes were immediately removed, placed in RNA extraction solution, and homogenized. RNA was recovered using GeneHunter and Ambion RiboPure total RNA kits. Samples of RNA were checked for purity by examination of the 28S and 18S ribosomal RNA bands from denaturing gel electrophoresis, by 260/280 ratios from scans with a Nanodrop ND 1000 spectrophotometer, and by readouts of the Agilent Bioanalyzer. Total RNA samples were prepared and hybridized to Affymetrix *Xenopus laevis* GeneChip Genome Arrays at the University of Texas Southwestern Medical Center Microarray Array Core Facility following standard Affymetrix protocols. Affymetrix Microarray Analysis Suite (MAS) v.5.0 was used to scan and process each microarray chip. The signals of quality control and poly(A) transcripts revealed that hybridizations were of high quality in all chips. Quality control probesets (i.e., spike in and housekeeping genes) were removed in subsequent statistical analyses. Non-unique probesets (i.e. interrogating different transcript variants from same genes) that represent <4% of the entire array were not masked out.

Hybridizing RNA from a heterospecific species to a microarray designed for a related species can have a dramatic impact on the signal recovered from microarrays [35]–[37]. Consequently, we directly assayed sequence divergence by hybridizing genomic DNA from *Xenopus laevis* and *X. muelleri* each separately to the Affymetrix *Xenopus laevis* GeneChip Genome Array. Genomic DNA was extracted from liver from eight individuals/species (four male and four female) using a QIAGEN DNeasy kit. Genomic DNA was quantified using a Nanodrop ND 1000 spectrophotometer and 2.5 µg/µl from each of the eight individuals was pooled to produce 20 µg genomic DNA for subsequent fragmentation. The 20 µg pooled genomic DNA was fragmented by a DNase I digestion using 2.5 µl 10× One-Phor-All Buffer (Amersham Biosciences), 0.015 U/µg DNase I (Amersham Biosciences), and 14.125 µl H_2_O to form a 25 µl reaction. Reactions were incubated at 37°C for 10 minutes and the DNase I was inactivated by incubating the reaction at 100°C for 10 minutes. Reactions were visualized on 2.5% agarose gels stained with SYBR Green to confirm that fragments were in the 50–200 bp size range necessary for hybridization to Affymetrix microarrays. Fragments were labeled on the 3′ termini and hybridized to the *X. laevis* Affymetrix Genome Array following standard protocols recommended by Affymetrix.

We used an electronic mask to eliminate probes that behaved poorly due to sequence divergence. To do so, we modified the Xspecies perl script (Version 1.1) in [146] to incorporate information gained by comparing the ratio of genomic hybridization intensity of *X. muelleri* to *X. laevis* for each probe pair. The Xspecies perl script selects a probe-set when one or more PM probe-pair (s) meets user-specified criteria of hybridization intensity and creates a new probe-definition file (.cdf) that contains only those probe-pairs that meet the user-specified criteria. This, in theory, should create a hybridization signal that interrogates gene expression in a less biased manner compared to not eliminating these probes. To conservatively guard against sequence divergence we set the lower ratio of hybridization intensity to 0.99 and explored variation in the number of probes eliminated at a variety of upper thresholds with the idea that higher intensity for *X. muelleri* is not as damaging compared to probes that have a hybridization signal lower for *X. muelleri* when compared to *X. laevis* (Fig. 6). In examining the threshold variation on the number of probesets remaining, we chose to analyze the .cdf generated at the 1.01 and 1.10 level. Both sets provided a similar general pattern but the effect was more prominent for the less stringent threshold. We report here the results generated using an upper threshold of 1.10 in subsequent analyses of gene expression which resulted in the removal of 226,841 individual probes and provided 11,485 probesets for further analysis.

### Data Analysis

Using the chip definition files created from the Xspecies perl script analyses, we conducted two separate comparisons to uncover patterns of differential expression between *Xenopus laevis* and hybrids, *X. muelleri* compared to hybrids, and *X. laevis* compared to *X. muelleri*. First, the *Xenopus laevis* and hybrid data (filtered with the Xspecies mask) were normalized using RMAexpress [147] with default parameters for background correction and quantile normalization. These RMA normalized data were then imported into the R statistical environment and tested for differences in expression between *X. laevis* and hybrids for each of the 11,485 genes using a moderated *t*-statistic based on an empirical Bayes method in the limma package found in Bioconductor [148]. The TopTable function was then used to output the FDR-adjusted *P*-values for differential expression. We normalized *X. muelleri* and hybrid chips together using RMA and repeated the analyses to uncover differential expression between *X. muelleri* and hybrids. Finally, we normalized *X. laevis* and *X. muelleri* chips together using RMA and repeated the analysis to uncover genes misexpressed between the two species. This later test was used to discover patterns of expression behavior in hybrids compared to interspecies expression behavior.

## Supporting Information

Table S1 Genes differentially expressed between *Xenopus laevis* and hybrids. Probe ID is the Affymetrix reference number for a particular probeset. Columns B–E are expression values after RMA normalization for *Xenopus laevis* and columns F–I are hybrids. MeanLaevis is the mean expression value of *X. laevis* and MeanHybrid is the mean expression value for hybrids. L-H is the fold change in expression (MeanLaevis - MeanHybrid). t is the moderated t-statistic. P.Value is the unadjusted P value obtained from the empirical Bayes function. adj.P.Val is the FDR corrected P value according to Benjamini and Hochberg (1995). B is the B-statistic which is the log-odds that a gene is differentially expressed. Description is the annotation information for a probeset as given by Affymetrix.
(0.09 MB XLS)

Table S2 Genes differentially expressed between *Xenopus muelleri* and hybrids. Probe ID is the Affymetrix reference number for a particular probeset. Columns B–D are expression values after RMA normalization for *Xenopus laevis* and columns E–H are hybrids. MeanMuelleri is the mean expression value of *X. muelleri* and MeanHybrid is the mean expression value for hybrids. M-H is the fold change in expression (MeanMuelleri - MeanHybrid). t is the moderated t-statistic. P.Value is the unadjusted P value obtained from the empirical Bayes function. adj.P.Val is the FDR corrected P value according to Benjamini and Hochberg (1995). B is the B-statistic which is the log-odds that a gene is differentially expressed. Description is the annotation information for a probeset as given by Affymetrix.
(2.19 MB XLS)

Table S3 Genes differentially expressed between *Xenopus muelleri* and *X. laevis*. Probe ID is the Affymetrix reference number for a particular probeset. Columns B–D are expression values after RMA normalization for *Xenopus muelleri* and columns E–H are *X. laevis*. MeanMuell is the mean expression value of *X. muelleri* and MeanLaev is the mean expression value for hybrids. M-L is the fold change in expression (MeanMuell - MeanLaev). t is the moderated t-statistic. P.Value is the unadjusted P value obtained from the empirical Bayes function. adj.P.Val is the FDR corrected P value according to Benjamini and Hochberg (1995). B is the B-statistic which is the log-odds that a gene is differentially expressed. Description is the annotation information for a probeset as given by Affymetrix.
(3.78 MB XLS)

Table S4 Top 30 candidate transcripts upregulated in *X. laevis* and differentially expressed between *X. laevis* and hybrid. Expression values are in log2 scale; SD = standard deviation of expression values. P values are adjusted according to FDR moderated t-tests.
(0.09 MB DOC)

Table S5 Top 30 candidate transcripts upregulated in hybrids and differentially expressed between *X. laevis* and hybrid. Expression values are in log2 scale; SD = standard deviation of expression values. P values are adjusted according to FDR moderated t-tests.
(0.09 MB DOC)

Table S6 Top 30 candidate transcripts upregulated in *X. muelleri* and differentially expressed between *X. muelleri* and hybrid. Expression values are in log2 scale; SD = standard deviation of expression values. P values are adjusted according to FDR moderated t-tests.
(0.09 MB DOC)

Table S7 Top 30 candidate transcripts upregulated in hybrids and differentially expressed between *X. muelleri* and hybrid. Expression values are in log2 scale; SD = standard deviation of expression values. P values are adjusted according to FDR moderated t-tests.
(0.09 MB DOC)

Table S8 Top 20 transcripts with the highest sequence conservation between Affymetrix probesets and *Xenopus tropicalis* genome. Probe ID is the Affymetrix reference number for a particular probeset. Columns B–C are mean differences between RMA-normalized expression values of *Xenopus laevis* and hybrids (B) and *Xenopus muelleri* and hybrids (C). Columns D and E are absolute values from columns B and C, respectively. Column F is mean % sequence identity of sequences between Affymetrix probesets and *Xenopus tropicalis* genome generated by BLAST. Column G is average alignment length from BLAST. Column H is average mismatch number of the alignments from F and G. Column I is mean E-value generated by BLAST. J column contains Affymetrix descriptions of transcripts.
(0.02 MB XLS)
