# Supplementary material for: Sterility and Gene Expression in Hybrid Males of Xenopus laevis and X. muelleri
Source: PLoS One. 2007 Aug 22;2(8):e781. doi: 10.1371/journal.pone.0000781 (PMC1940320; doi:10.1371/journal.pone.0000781)
Supplement: Table S4 — Top 30 candidate transcripts upregulated in X. laevis and differentially expressed between X. laevis and hybrid. Expression values are in log2 scale; SD = standard deviation of expression values. P values are adjusted according to FDR moderated t-tests. (0.09 MB DOC) [file pone.0000781.s004.doc]

Table S4.

| **ProbeID** | **GeneBank ID** | **Target Gene** | **Gene Symbol** | **Description/Molecular Function** | **Mean Laev.** | **SD Laev.** | **Mean Hybrid** | **SD Hybrid** | **L-H** | ***P* Value** |
| --- | --- | --- | --- | --- | --- | --- | --- | --- | --- | --- |
| Xl.22458.1.A1_at | BF072333 | ESTs |  |  | 9.905 | 1.226 | 6.673 | 0.685 | 3.232 | 0.0393 |
| Xl.14330.2.S1_at | BG021901 | ESTs | MGC85135 | Moderately similar to Myc-interacting zinc finger protein (H.sapiens) | 6.920 | 0.367 | 3.707 | 0.630 | 3.213 | 0.0053 |
| Xl.14584.1.A1_at | BJ091208 | ESTs |  |  | 11.721 | 0.426 | 9.118 | 1.086 | 2.604 | 0.0405 |
| Xl.5076.1.S1_at | BG486062 | ESTs | LOC495423 | Protein amino acid phosphorylation, nucleotide binding and kinase activity | 7.099 | 0.839 | 4.697 | 0.525 | 2.403 | 0.0390 |
| Xl.13064.1.A1_at | BJ097646 | ESTs |  |  | 8.371 | 0.546 | 5.992 | 0.905 | 2.379 | 0.0408 |
| Xl.13044.1.S1_at | BU912107 | ESTs |  | Weakly similar to nucleolar phosphoprotein Nopp34 (Homo sapiens) | 8.263 | 0.492 | 5.985 | 0.838 | 2.278 | 0.0405 |
| Xl.2583.1.S1_at | BJ045215 | ESTs | LOC495201 |  | 11.941 | 0.382 | 9.701 | 0.958 | 2.240 | 0.0459 |
| Xl.8438.1.A1_at | BF072047 | ESTs | MGC84983 | Transcription factor TFIIE complex, RNA pol II transcription factor | 8.833 | 0.600 | 6.716 | 0.641 | 2.117 | 0.0405 |
| Xl.553.1.S1_at | AF183394.1 | Casein kinase I | CKIe | Protein amino acid phosphorylation, nucleotide binding and kinase activity | 8.194 | 0.286 | 6.101 | 0.649 | 2.093 | 0.0269 |
| Xl.13889.1.A1_at | BJ077199 | ESTs |  |  | 7.259 | 0.251 | 5.332 | 0.289 | 1.926 | 0.0084 |
| Xl.4752.1.A1_at | BJ057383 | ESTs | MGC81145 | Weakly similar to tubulin-specific chaperone B (H.sapiens) | 5.399 | 0.539 | 3.530 | 0.547 | 1.869 | 0.0405 |
| Xl.10826.1.A1_at | CB560349 | ESTs | MGC82938 | Moderately similar to 5-AMP-activated protein kinase (H.sapiens) | 8.377 | 0.292 | 6.514 | 0.700 | 1.863 | 0.0405 |
| Xl.3637.1.S1_at | BF071889 | Zinc finger | zmat2 | Nucleic acid binding | 8.729 | 0.604 | 6.911 | 0.300 | 1.818 | 0.0384 |
| Xl.11414.1.A1_at | BG552599 | ESTs |  |  | 6.850 | 0.471 | 5.033 | 0.509 | 1.817 | 0.0399 |
| Xl.17907.1.A1_s_at | BQ735473 | Glycoprotein | gpm6a-A |  | 6.511 | 0.388 | 4.710 | 0.670 | 1.801 | 0.0455 |
| Xl.9507.1.A1_at | BJ090330 | ESTs | MGC84594 | Protein disulfide oxidoreductase activity | 7.776 | 0.518 | 6.031 | 0.465 | 1.745 | 0.0405 |
| Xl.23530.1.S1_at | BC044077.1 | MGC52668 |  |  | 8.046 | 0.136 | 6.326 | 0.334 | 1.720 | 0.0151 |
| Xl.2955.1.A1_at | BJ091076 | ESTs |  | Weakly similar to and SOCS box-containing protein 2 (Homo sapiens) | 7.606 | 0.397 | 5.919 | 0.261 | 1.688 | 0.0256 |
| Xl.18999.1.A1_at | BI449483 | RALDH2 | ALDH1A2 | Oxidoreductase activity | 8.074 | 0.332 | 6.400 | 0.313 | 1.674 | 0.0248 |
| Xl.901.1.S1_s_at | D42060.1 | Pleiotrophic factor | MGC84465 | Regulation of progression through cell cycle | 5.050 | 0.656 | 3.377 | 0.247 | 1.673 | 0.0458 |
| Xl.6988.1.A1_at | BI349938 | ESTs | MGC82200 |  | 9.027 | 0.353 | 7.370 | 0.397 | 1.658 | 0.0320 |
| Xl.24473.2.S1_at | BE679976 | ESTs | LOC494660 |  | 4.628 | 0.547 | 2.991 | 0.323 | 1.636 | 0.0405 |
| Xl.15612.1.A1_at | BJ079143 | ESTs |  |  | 7.059 | 0.500 | 5.423 | 0.407 | 1.636 | 0.0408 |
| Xl.15671.1.S1_at | BJ056235 | ESTs |  |  | 5.053 | 0.445 | 3.461 | 0.479 | 1.592 | 0.0459 |
| Xl.11452.1.S1_at | BF025643 | ESTs |  |  | 8.763 | 0.223 | 7.174 | 0.383 | 1.590 | 0.0269 |
| Xl.2688.1.S1_at | BG161445 | ESTs | MGC68790 | Catalytic activity | 11.036 | 0.549 | 9.465 | 0.148 | 1.571 | 0.0405 |
| Xl.5907.1.A1_at | BJ090062 | ESTs |  | Weakly similar to GAP-associated tyrosine phosphoprotein p62 (H.sapiens) | 9.354 | 0.471 | 7.807 | 0.397 | 1.547 | 0.0454 |
| Xl.10407.1.A1_at | BF072385 | ESTs |  | Moderately similar to sel-1 suppressor of lin-12 (Homo sapiens) | 7.505 | 0.136 | 5.962 | 0.456 | 1.543 | 0.0332 |
| Xl.26339.1.A1_at | BM261816 | ESTs | MGC114836 |  | 5.232 | 0.538 | 3.718 | 0.228 | 1.514 | 0.0432 |
| Xl.23438.1.S1_at | BF024995 | ESTs | LOC398494 | Similar to heterogeneous nuclear ribonucleoprotein U | 10.609 | 0.404 | 9.108 | 0.125 | 1.501 | 0.0301 |
