# Supplementary material for: Sterility and Gene Expression in Hybrid Males of Xenopus laevis and X. muelleri
Source: PLoS One. 2007 Aug 22;2(8):e781. doi: 10.1371/journal.pone.0000781 (PMC1940320; doi:10.1371/journal.pone.0000781)
Supplement: Table S5 — Top 30 candidate transcripts upregulated in hybrids and differentially expressed between X. laevis and hybrid. Expression values are in log2 scale; SD = standard deviation of expression values. P values are adjusted according to FDR moderated t-tests. (0.09 MB DOC) [file pone.0000781.s005.doc]

Table S5.

| **ProbeID** | **GeneBank ID** | **Target Gene** | **Gene Symbol** | **Description/Molecular Function** | **Mean Laev.** | **SD Laev.** | **Mean Hybrid** | **SD Hybrid** | **L-H** | ***P* Value** |
| --- | --- | --- | --- | --- | --- | --- | --- | --- | --- | --- |
| Xl.831.1.S1_s_at | L11294.1 | neuropeptide Y | NPY | Hormone activity | 2.954 | 0.464 | 6.966 | 0.907 | -4.013 | 0.0053 |
| Xl.24869.1.A1_at | BE026534 | ESTs |  |  | 4.874 | 1.235 | 8.633 | 0.781 | -3.758 | 0.0269 |
| Xl.233.1.S1_s_at | AF035016.1 | synaptobrevin | xsybi | Vesicle-mediated transport | 3.417 | 0.336 | 7.016 | 1.506 | -3.600 | 0.0383 |
| Xl.8916.1.S1_at | AW766707 | ESTs |  |  | 3.883 | 1.180 | 7.225 | 0.387 | -3.342 | 0.0256 |
| Xl.21956.1.S1_at | BC042271.1 | MGC53461 |  |  | 6.978 | 0.330 | 10.026 | 0.178 | -3.048 | 0.0016 |
| Xl.24635.1.S1_at | CB561521 | MGC86242 |  |  | 4.239 | 0.890 | 7.280 | 0.691 | -3.041 | 0.0268 |
| Xl.24583.1.S1_at | BU904046 | ESTs | LOC398631 | Oxidoreductase activity | 3.000 | 0.287 | 6.016 | 0.579 | -3.016 | 0.0053 |
| Xl.262.1.S1_at | D10259.1 | Homeodomain for Distal-less | distalless | regulation of transcription, DNA-dependent | 4.221 | 0.787 | 7.189 | 0.731 | -2.969 | 0.0256 |
| Xl.3958.1.A1_at | BF071965 | ESTs |  |  | 7.515 | 0.435 | 10.445 | 0.471 | -2.929 | 0.0053 |
| Xl.16776.3.A1_at | BJ056076 | ESTs | MGC68565 | Protein binding | 6.005 | 0.247 | 8.865 | 0.358 | -2.860 | 0.0027 |
| Xl.3481.1.S1_at | BF612780 | ESTs |  | Highly similar to ubiquitin-like 5 (Homo sapiens) | 3.683 | 0.453 | 6.290 | 1.104 | -2.607 | 0.0418 |
| Xl.25297.1.S1_at | BG020931 | ESTs |  |  | 4.007 | 0.587 | 6.433 | 0.562 | -2.426 | 0.0234 |
| Xl.11954.1.S1_at | AJ277826.1 | PAK5 protein | PAK5 | Protein amino acid phosphorylation | 6.897 | 0.358 | 9.269 | 0.326 | -2.373 | 0.0053 |
| Xl.252.1.S1_s_at | AJ224125.1 | erg | erg-A; erg-B | Transcription factor activity; sequence-specific DNA binding | 3.272 | 0.516 | 5.570 | 0.872 | -2.298 | 0.0408 |
| Xl.26415.1.S1_at | CD361045 | ESTs |  | Weakly similar to testis expressed protein isoform (Mus musculus) | 6.598 | 0.441 | 8.887 | 0.151 | -2.289 | 0.0056 |
| Xl.23573.1.S1_at | BC041496.1 | Similar to thymine-DNA glycosylase | TDG | DNA repair; hydrolase activity, acting on glycosyl bonds | 3.862 | 0.882 | 6.142 | 0.321 | -2.280 | 0.0399 |
| Xl.21661.1.S1_at | AF526274.1 | T-box transcription factor | Tbx1 | Regulation of transcription, DNA-dependent | 4.817 | 0.643 | 7.096 | 0.283 | -2.279 | 0.0188 |
| Xl.22343.1.A1_at | BG020671 | ESTs |  |  | 5.736 | 0.797 | 8.007 | 0.586 | -2.271 | 0.0405 |
| Xl.6132.3.S1_a_at | CB592496 | MGC114734 |  | Moderately similar to serinethreonine protein phosphatase (H.sapiens) | 5.016 | 0.904 | 7.264 | 0.510 | -2.248 | 0.0460 |
| Xl.8467.1.S1_at | BC043743.1 | ATPase | atp1a3 | ATPase activity, coupled to transmembrane movement of ions | 5.046 | 0.461 | 7.288 | 0.168 | -2.242 | 0.0069 |
| Xl.2789.1.A1_at | BJ091236 | ESTs |  | Weakly similar to putative lymphocyte G0G1 switch protein 2 (H.sapiens) | 4.109 | 0.675 | 6.347 | 0.795 | -2.237 | 0.0470 |
| Xl.14349.1.S1_at | BJ099365 | ESTs | MGC132021 | Acyl-CoA binding | 7.953 | 0.365 | 10.174 | 0.292 | -2.221 | 0.0067 |
| Xl.12561.1.S1_at | BJ041426 | XMeis1-3 | LOC398167 | Homeodomain transcription factor | 5.561 | 0.817 | 7.764 | 0.446 | -2.202 | 0.0405 |
| Xl.8719.1.S1_at | CD302009 | dynein light chain 2 | dynll2 | Microtubule motor activity | 7.985 | 0.333 | 10.176 | 0.238 | -2.191 | 0.0053 |
| Xl.5630.1.S1_x_at | AF549947.1 | MGC131089 |  | RNA binding | 6.624 | 0.834 | 8.788 | 0.303 | -2.164 | 0.0403 |
| Xl.20475.1.S1_at | BQ730542 | ESTs |  |  | 3.535 | 0.060 | 5.677 | 0.909 | -2.142 | 0.0405 |
| Xl.24218.1.S1_at | CB943601 | ESTs | Cnef01 | Weakly similar to apolipoprotein C-I precursor (H.sapiens) | 8.974 | 0.878 | 11.080 | 0.402 | -2.105 | 0.0468 |
| Xl.11050.2.A1_at | BE027068 | ESTs | MGC80999 |  | 7.291 | 0.506 | 9.389 | 0.231 | -2.098 | 0.0152 |
| Xl.19741.1.S1_at | BQ398596 | ESTs | LOC495153 |  | 5.052 | 0.686 | 7.137 | 0.245 | -2.085 | 0.0281 |
| Xl.16301.1.A1_at | BM179219 | ESTs |  |  | 2.843 | 0.401 | 4.927 | 0.408 | -2.084 | 0.0160 |
