# Supplementary material for: Sterility and Gene Expression in Hybrid Males of Xenopus laevis and X. muelleri
Source: PLoS One. 2007 Aug 22;2(8):e781. doi: 10.1371/journal.pone.0000781 (PMC1940320; doi:10.1371/journal.pone.0000781)
Supplement: Table S6 — Top 30 candidate transcripts upregulated in X. muelleri and differentially expressed between X. muelleri and hybrid. Expression values are in log2 scale; SD = standard deviation of expression values. P values are adjusted according to FDR moderated t-tests. (0.09 MB DOC) [file pone.0000781.s006.doc]

Table S6.

| **ProbeID** | **GeneBank ID** | **Target Gene** | **Gene Symbol** | **Description/Molecular Function** | **Mean Muell.** | **SD Muell.** | **Mean Hybrid** | **SD Hybrid** | **M-H** | ***P* Value** |
| --- | --- | --- | --- | --- | --- | --- | --- | --- | --- | --- |
| Xl.22458.1.A1_at | BF072333 | ESTs |  |  | 10.466 | 1.175 | 6.230 | 0.705 | 4.236 | 0.0015 |
| Xl.4588.1.A1_at | BJ098745 | ESTs | Ube2e2 | Ubiquitin-protein ligase activity | 6.870 | 0.026 | 3.355 | 0.417 | 3.515 | 0.0002 |
| Xl.21527.1.S1_at | CA791375 | Phosphoinositide-3-kinase | pik3ca-A | Phosphatidylinositol 3-kinase activity | 7.225 | 0.600 | 3.898 | 0.552 | 3.326 | 0.0009 |
| Xl.11477.1.S1_at | CB561158 | ESTs |  |  | 8.790 | 1.079 | 5.544 | 0.304 | 3.246 | 0.0022 |
| Xl.20548.1.A1_at | BQ736300 | ESTs |  |  | 6.833 | 1.683 | 3.629 | 0.511 | 3.204 | 0.0107 |
| Xl.544.1.S1_at | AF170275.1 | Occludin | LOC398133 |  | 6.238 | 0.359 | 3.051 | 0.263 | 3.187 | 0.0002 |
| Xl.801.1.A1_at | M63872.1 | Goosecoid | goosecoid | Regulation of transcription | 5.982 | 1.928 | 2.964 | 0.252 | 3.018 | 0.0175 |
| Xl.19995.1.S1_at | BJ065331 | Spectrin (alpha-fodrin) | sptan1-A | Cytoskeleton formation | 6.693 | 0.241 | 3.716 | 0.189 | 2.977 | 0.0002 |
| Xl.2956.1.S1_at | BG022603 | Elastase | ela3b | Serine-type endopeptidase activity | 8.423 | 1.610 | 5.484 | 0.521 | 2.940 | 0.0131 |
| Xl.7311.1.A1_at | BG553598 | ESTs | MGC82349 | Moderately similar to DHHC1 protein (Homo sapiens) | 7.025 | 0.411 | 4.107 | 0.295 | 2.918 | 0.0005 |
| Xl.8231.1.A1_at | AW148242 | ESTs |  |  | 8.475 | 1.944 | 5.572 | 0.436 | 2.903 | 0.0225 |
| Xl.21454.1.A1_at | X65256.1 | Transmembrane conductance regulator | cftr-A | ATPase activity, coupled to transmembrane movement of substances | 6.306 | 1.227 | 3.415 | 0.265 | 2.891 | 0.0053 |
| Xl.7631.1.A2_at | BG371327 | LOC398509 | LOC398509 |  | 7.868 | 0.259 | 4.989 | 0.388 | 2.879 | 0.0005 |
| Xl.18472.1.A1_at | BI347887 | ESTs |  |  | 8.657 | 0.470 | 5.801 | 0.366 | 2.856 | 0.0007 |
| XlAffx.1.5.S1_at | M63663.1 | X.borealis B1 protein | B1 | Transcription regulation | 10.151 | 1.738 | 7.301 | 0.341 | 2.849 | 0.0161 |
| Xl.25724.1.S1_at | AB000736.1 | Myelin basic protein | Mbp | Structural constituent of myelin sheath | 6.481 | 0.806 | 3.643 | 0.423 | 2.839 | 0.0023 |
| Xl.17719.1.A1_at | BG811302 | ESTs |  |  | 6.435 | 1.594 | 3.599 | 0.592 | 2.836 | 0.0154 |
| Xl.13920.1.A1_at | BG233631 | ESTs |  |  | 7.891 | 0.270 | 5.057 | 1.100 | 2.834 | 0.0072 |
| Xl.23289.1.S1_at | BC046655.1 | MGC52879 | MGC52879 |  | 5.725 | 1.563 | 2.897 | 0.327 | 2.828 | 0.0120 |
| Xl.24429.1.S1_at | CB563855 | ESTs |  |  | 9.142 | 0.695 | 6.333 | 1.243 | 2.809 | 0.0139 |
| Xl.18120.1.S1_at | BG885943 | ESTs |  |  | 5.914 | 1.333 | 3.110 | 0.425 | 2.805 | 0.0085 |
| Xl.14265.1.A1_at | BM191733 | ESTs |  |  | 8.792 | 0.273 | 5.995 | 0.529 | 2.796 | 0.0010 |
| Xl.8670.1.A1_at | AW159159 | ESTs |  |  | 8.253 | 0.644 | 5.462 | 0.519 | 2.791 | 0.0021 |
| Xl.19899.1.A1_at | BQ400337 | ESTs |  | Moderately similar to bromodomain 2B (Homo sapiens) | 6.275 | 0.569 | 3.514 | 0.222 | 2.760 | 0.0008 |
| Xl.1029.1.S1_at | U38225.1 | Glutamate decarboxylase | gad1-A | Carboxy-lyase activity | 7.783 | 0.537 | 5.026 | 1.173 | 2.757 | 0.0113 |
| Xl.8437.1.A1_at | BJ097670 | ESTs |  | Moderately similar to serinethreonine kinase (Homo sapiens) | 7.380 | 0.284 | 4.628 | 0.569 | 2.752 | 0.0013 |
| Xl.298.1.S1_at | AF079559.1 | Homeobox protein BIX1 | Bix1 | Regulation of transcription, DNA-dependent | 6.277 | 1.266 | 3.530 | 0.266 | 2.747 | 0.0070 |
| Xl.990.1.S1_at | U09769.1 | Achaete-scute homolog | ash3b-A | Transcription regulator activity | 6.100 | 1.034 | 3.365 | 0.449 | 2.736 | 0.0051 |
| Xl.2789.1.A1_at | BJ091236 | ESTs |  | Weakly similar to putative lymphocyte G0G1 switch protein 2 (H.sapiens) | 8.615 | 0.953 | 5.900 | 0.810 | 2.716 | 0.0085 |
| Xl.9600.1.A1_at | BG730683 | ESTs |  |  | 5.580 | 1.070 | 2.879 | 0.386 | 2.701 | 0.0054 |
