# Supplementary material for: Sterility and Gene Expression in Hybrid Males of Xenopus laevis and X. muelleri
Source: PLoS One. 2007 Aug 22;2(8):e781. doi: 10.1371/journal.pone.0000781 (PMC1940320; doi:10.1371/journal.pone.0000781)
Supplement: Table S7 — Top 30 candidate transcripts upregulated in hybrids and differentially expressed between X. muelleri and hybrid. Expression values are in log2 scale; SD = standard deviation of expression values. P values are adjusted according to FDR moderated t-tests. (0.09 MB DOC) [file pone.0000781.s007.doc]

Table S7.

| **ProbeID** | **GeneBank ID** | **Target Gene** | **Gene Symbol** | **Description/Molecular Function** | **Mean Muell.** | **SD Muell.** | **Mean Hybrid** | **SD Hybrid** | **M-H** | ***P* value** |
| --- | --- | --- | --- | --- | --- | --- | --- | --- | --- | --- |
| Xl.21811.1.S1_at | BC041228.1 | GKAP42 | gkap42 | Protein kinase | 2.938 | 0.502 | 11.273 | 0.361 | -8.336 | 0.0000 |
| Xl.3208.1.A1_at | BJ057240 | ESTs | MGC115620 | Exonuclease activity | 2.724 | 0.378 | 10.532 | 0.236 | -7.808 | 0.0000 |
| Xl.24746.1.S1_at | BF049564 | ESTs |  | Weakly similar to microsomal glutathione S-transferase 3 (H.sapiens) | 4.310 | 0.808 | 11.573 | 0.254 | -7.263 | 0.0000 |
| Xl.2458.1.A1_at | BJ085862 | ESTs | LOC496017 |  | 3.089 | 0.635 | 10.287 | 0.512 | -7.198 | 0.0000 |
| Xl.2565.1.S1_at | BC044001.1 | Similar to alpha-Tubulin | MGC53359 | GTPase activity | 3.141 | 0.963 | 10.331 | 0.571 | -7.190 | 0.0001 |
| Xl.5466.1.A1_at | BJ087266 | ESTs | LOC445827 | Histidine-tRNA ligase activity | 4.455 | 1.418 | 11.379 | 0.232 | -6.924 | 0.0002 |
| Xl.5149.1.S1_at | CB592843 | ESTs |  |  | 5.214 | 0.317 | 12.032 | 0.095 | -6.818 | 0.0000 |
| Xl.16507.1.A1_at | BJ076999 | ESTs | LOC495063 | Vesicle-mediated transport | 2.695 | 0.547 | 9.318 | 0.159 | -6.623 | 0.0000 |
| Xl.3862.2.S1_x_at | CD361360 | Translation factor sui1 homolog | gc20 | Translation initiation factor activity | 2.985 | 0.562 | 9.407 | 0.387 | -6.423 | 0.0000 |
| Xl.7933.1.S1_at | L06232.1 | Beta-Tubulin | betatub56d | GTPase activity | 6.534 | 0.488 | 12.946 | 0.258 | -6.412 | 0.0000 |
| Xl.7482.2.A1_x_at | BU908393 | MGC53533 | MGC53533 | Ubiquitin-protein ligase activity | 2.950 | 0.847 | 9.295 | 0.433 | -6.345 | 0.0001 |
| Xl.2855.1.A1_at | BM191868 | ESTs | MGC82859 |  | 3.937 | 1.076 | 10.276 | 0.234 | -6.340 | 0.0001 |
| Xl.2839.1.S1_at | BC041270.1 | Protein translocation complex | sec61beta |  | 5.554 | 0.611 | 11.866 | 0.229 | -6.312 | 0.0000 |
| Xl.24180.1.A1_at | BG552332 | ESTs | MGC81070 | Signalosome complex | 4.293 | 1.057 | 10.557 | 0.181 | -6.263 | 0.0001 |
| Xl.3536.2.S1_x_at | BF615663 | ESTs | LOC495200 | Highly similar to transcription factor BTF3a (H.sapiens) | 4.740 | 0.800 | 10.999 | 0.582 | -6.259 | 0.0001 |
| Xl.702.1.S1_a_at | J03167.1 | cyclin B2 | C-B2 | Regulation of progression through cell cycle | 2.765 | 0.540 | 8.955 | 0.548 | -6.190 | 0.0001 |
| Xl.8046.1.S1_at | AW198888 | ESTs | MGC68738 | Hydrogen-transporting ATP synthase activity | 3.636 | 1.006 | 9.797 | 0.477 | -6.161 | 0.0002 |
| Xl.1079.1.S1_at | S64727.1 | Ferritin | Ferritin | Ferroxidase activity | 6.126 | 0.938 | 12.213 | 0.183 | -6.087 | 0.0001 |
| Xl.11981.1.S1_at | BE678238 | ESTs | LOC495086 | Catalytic activity; ATP binding | 3.446 | 1.086 | 9.516 | 0.449 | -6.070 | 0.0002 |
| Xl.23752.2.S1_at | BJ051838 | Ribosomal protein S3a | rps3a | Structural constituent of ribosome | 5.653 | 1.594 | 11.717 | 0.457 | -6.064 | 0.0005 |
| Xl.7947.1.A1_at | BJ082612 | ESTs |  |  | 4.405 | 0.715 | 10.469 | 0.323 | -6.064 | 0.0001 |
| Xl.19459.1.S1_at | BQ385898 | ESTs | MGC82659 |  | 3.991 | 1.137 | 10.009 | 0.847 | -6.018 | 0.0004 |
| Xl.14065.1.A1_at | AW147826 | ESTs |  |  | 3.293 | 1.216 | 9.276 | 0.295 | -5.983 | 0.0002 |
| Xl.6585.1.S1_at | BJ080015 | HIV-1 rev binding protein 2 | HRB2 | RNA binding | 3.246 | 0.734 | 9.214 | 0.420 | -5.968 | 0.0001 |
| Xl.5160.1.S1_at | BJ057196 | HMG-box protein | HMG2L1 | Regulation of transcription, DNA-dependent | 2.789 | 0.072 | 8.738 | 1.408 | -5.950 | 0.0006 |
| Xl.25536.1.A1_at | BE677987 | ESTs |  |  | 4.328 | 0.821 | 10.255 | 0.499 | -5.927 | 0.0001 |
| Xl.25863.1.A1_s_at | BG554560 | ESTs | LOC495205 |  | 5.476 | 0.623 | 11.380 | 0.253 | -5.904 | 0.0001 |
| Xl.5904.1.S1_x_at | BJ049960 | ESTs | MGC114751 | Intracellular protein transport | 2.742 | 0.331 | 8.621 | 0.566 | -5.879 | 0.0001 |
| Xl.11443.1.S1_at | BC041251.1 | AP-3 | ap3s1 | Coated vesicle membrane | 3.634 | 0.689 | 9.485 | 0.549 | -5.851 | 0.0001 |
| Xl.4839.1.S1_at | CD324878 | H1 histone | h1fx |  | 3.604 | 0.924 | 9.444 | 0.334 | -5.840 | 0.0001 |
